# Tumor Infiltrating Lymphocyte (TIL) Therapy for Solid Tumor Treatment: Progressions and Challenges

**DOI:** 10.3390/cancers14174160

**Published:** 2022-08-27

**Authors:** Yueshui Zhao, Jian Deng, Shuangfeng Rao, Sipeng Guo, Jing Shen, Fukuan Du, Xu Wu, Yu Chen, Mingxing Li, Meijuan Chen, Xiaobing Li, Wanping Li, Li Gu, Yuhong Sun, Zhuo Zhang, Qinglian Wen, Zhangang Xiao, Jing Li

**Affiliations:** 1 Laboratory of Molecular Pharmacology, Department of Pharmacology, School of Pharmacy, Southwest Medical University, Luzhou 646000, China; 2 Cell Therapy & Cell Drugs of Luzhou Key Laboratory, Southwest Medical University, Luzhou 646000, China; 3 South Sichuan Institute of Translational Medicine, Luzhou 646000, China; 4 Department of Pharmacy, The Affiliated Traditional Chinese Medicine Hospital of Southwest Medical University, Luzhou 646000, China; 5 Department of Oncology, The Affiliated Hospital of Southwest Medical University, Luzhou 646000, China; 6 Department of Oncology and Hematology, The Affiliated Traditional Chinese Medicine Hospital of Southwest Medical University, Luzhou 646000, China

**Keywords:** immunotherapy, tumor infiltrating lymphocytes (TIL), solid tumor

## Abstract

**Simple Summary:**

Over the past decade, cell-based immunotherapy has become a powerful strategy in solid cancer therapy. Tumor-infiltrating lymphocytes (TILs) are a group of intratumor lymphocytes. With the development of new technologies, the isolation and generation of TILs from tumor tissues have improved. Clinical use of TILs for solid tumor treatment showed good efficacy. In this review, we summarize the current strategies and challenges of TIL generation. We highlight the clinical trials where TIL therapy is used independently and in combination with other therapies for solid tumor treatment. Finally, the limitations, future potential, and directions of TIL therapy for solid tumor treatment are also discussed.

**Abstract:**

Over the past decade, immunotherapy, especially cell-based immunotherapy, has provided new strategies for cancer therapy. Recent clinical studies demonstrated that adopting cell transfer of tumor-infiltrating lymphocytes (TILs) for advanced solid tumors showed good efficacy. TIL therapy is a type of cell-based immunotherapy using the patient’s own immune cells from the microenvironment of the solid tumor to kill tumor cells. In this review, we provide a comprehensive summary of the current strategies and challenges in TIL isolation and generation. Moreover, the current clinical experience of TIL therapy is summarized and discussed, with an emphasis on lymphodepletion regimen, the use of interleukin-2, and related toxicity. Furthermore, we highlight the clinical trials where TIL therapy is used independently and in combination with other types of therapy for solid cancers. Finally, the limitations, future potential, and directions of TIL therapy for solid tumor treatment are also discussed.

## 1. Introduction

In the past few decades, immunotherapy has been the focus of cancer research and various methods have emerged to treat cancers, such as immune checkpoint inhibitors (ICI) against the programmed cell death protein-1 (PD-1) or its ligand (PD-L1) and cytotoxic T lymphocyte associated antigen-4 (CTLA-4), vascular endothelial growth factor (VEGF) inhibitors, cytokines, vaccines, and adoptive cellular therapy (ACT). The primary form of ACT is chimeric antigen receptor (CAR) T-cell therapy. In this strategy, T cells are obtained from the patient’s own peripheral blood cells and are modified to express a receptor that binds to the tumor antigen. CAR-T cell therapy has been approved for the treatment of some patients with hematological malignancies, such as lymphoblastic leukemia and B cell lymphoma [1]. However, currently CAR-T cell therapy has not been proven to be effective in the treatment of solid tumors. Therefore, in order to benefit patients with various types of solid tumors, it is necessary to award further research to TIL therapy which precedes CAR-T cell therapy. In addition, TIL therapy is expected to solve the problem of drug resistance of solid tumors to PD-1 antibodies.

The TIL-ACT process begins by isolating the natural infiltrating lymphocytes from the tumor tissues, expanding them in vitro, and then infusing these cells with a high dose (HD) of IL-2 into patients to identify and kill tumor cells. Prior to infusion of the TIL products, patients receive a non-myeloablative (NMA) lymphodepletion regimen. Pioneering work in the TIL-ACT field has been undertaken by the research group of Dr. Steven Rosenberg. In 1986, Rosenberg et al. isolated TILs from murine tumors and amplified them alongside interleukin-2 (IL-2) in vitro. They then infused them back into the tumor-bearing hosts, which caused significant antitumor reactivity [2]. In addition, they found that the combination of IL-2 with the infusion of TILs could improve the therapeutic effect [3]. Rosenberg then applied TIL therapy to patients with melanoma and published the first report on the successful treatment of melanoma with TIL-ACT in 1988 [4]. At present, an increasing number of clinical centers worldwide have implemented TIL-ACT, and this therapy has shown impressive results in patients with metastatic melanoma [5,6]. Significantly, TIL therapy is also applicable to other solid tumors to a certain extent, and has achieved good responses [7,8,9,10,11,12]. In this review, we will provide an overview of the current status of TIL therapy in solid tumors, and focus on the preparation process of TIL, strategies of screening effective TILs, and treatment protocols, as well as the application of combined therapy with TILs.

## 2. Current Clinical State of TIL Therapy in Melanoma

Since Rosenberg and his colleagues successfully applied TIL therapy to patients with metastatic melanoma, a series of clinical trials have emerged. Dafni et al. reported that the objective response rate (ORR) of advanced cutaneous melanoma patients who received the treatment which combined TIL-ACT with IL-2 from 1988 to 2016 was 41%, and the overall complete response rate (CRR) was 12% [13]. In addition, the ORR and CRR of patients who received the regimen of high dose (HD) IL-2 was 43% and 14%, respectively, compared with 35% and 7% in the low dose (LD) IL-2 group [13]. This analysis ruled out patients with uveal melanoma. TIL therapy has been successful when applied to the rare and refractory uveal melanoma and was first reported in 2017 [14]. The complete response (CR) and partial response (PR) of patients who received TIL and HD IL-2 was 4.5% and 31.8%, respectively [14]. In 2021, Iovance successfully carried out phase II trials of TIL product Lifileucel (LN-144) for patients with advanced melanoma after progression on ICI [15]. Patients received a mean number of TILs of 2.73 × 10^10^, and the disease control rate (DCR) was 80%, the ORR was 36%, the CR was 3%, and the PR was 33%. Remarkably, these results indicate that TIL therapy is a potential new treatment for patients with advanced melanoma after progression on ICI. More recently, the observed objective clinical response rate in patients with advanced melanoma was 50%, including two complete response and three partial response patients [16]. At present, TIL therapy is being tested mainly as a second-line treatment, and melanoma is still the main type of tumor in most clinical trials (Table 1).

## 3. Successful Application of TIL Therapy in Other Solid Tumors

In addition to melanoma, TIL therapy has been proven to have impressive clinical benefits for patients with cervical cancer [8,17,18], and has also shown preliminary efficacy in colorectal cancer [10], cholangiocarcinoma [9], non-small cell lung cancer [11], and breast cancer [7] (Table 2).

Persistent infection with human papilloma viruses (HPVs) is essential in the pathogenesis of virtually all cervical cancers [19]. A clinical study was designed to determine whether the ACT with HPV-TILs can mediate the regression of metastatic cervical cancer, and nine eligible cervical patients were recruited [8]. The HPV-TILs were initiated and selected based on their reactivity against HPV E6 and E7. Patients received a single-dose infusion of HPV-TIL followed by a high dose of IL-2. Three patients experienced an objective tumor response, two patients had complete responses lasting over one year, and the rest had partial responses. Rosenberg et al. further screened tumor-specific antigens through a personalized immunogenomic approach including whole-exome sequencing [20]. In addition, they also conducted a phase II clinical trial to evaluate the efficacy of TIL therapy in the treatment of metastatic human HPV-associated cancers [17]. Although the overall response rate was modest, the results support the premise that TIL-ACT can mediate tumor regression, which provides a wealth of potential for the treatment of metastatic human HPV-associated cancers.

Remarkably, in a phase two clinical trial, the TIL product of LN-145 successfully treated patients with advanced cervical cancer. Patients received a mean number of TILs of 2.8 × 10^10^; the ORR was 44%, including one complete response, nine partial responses, and two unconfirmed partial responses [18]. To this end, the Food and Drug Administration (FDA) awarded the status of breakthrough treatment to LN-145. Ultimately, this pivotal study has demonstrated the feasibility and efficacy of this approach in patients with cervical cancer and will ensure further development of adoptive cell therapy in these kinds of malignancies.

In 2014, Tran et al. successfully amplified neoantigen-specific TILs from a patient with metastatic cholangiocarcinoma [9]. After the patient received an infusion of IL-2 and 42.4 billion TILs, which contained a large number of CD4^+^ neoantigen-specific T cells, the patient achieved dramatic regression of liver and lung metastases [9].

In a phase two clinical trial (NCT01174121), a 50-year old woman with metastatic colorectal cancer received a single infusion of 1.48 × 10^11^ TILs consisting of approximately 75% CD8^+^ T cells, which can specifically recognize the KRAS G12D mutant [10]. All metastatic lesions in this patient achieved regression and they achieved a nine-month partial response.

In 2017, Lee et al. successfully isolated and amplified TILs in vitro from patients with breast cancer which showed their reaction to autologous tumor cells [21]. Zacharakis et al. administrated TIL therapy to patients with hormone receptor-positive metastatic breast cancer in 2018 [7]. In short, the patient underwent complete and durable tumor regression after receiving a combination treatment of TIL therapy and anti-PD-1 monoclonal antibodies. It is worth noting that they screened out the TILs that were mainly CD4^+^ T cells (62.5%), which could recognize the mutant of four proteins (SLC3A2, KIAA0368, CADPS2, and CTSB) [7].

In 2020, it was reported that the combination of PD-1 inhibitor and TIL therapy showed preliminary efficacy in the treatment of NSCLC [11]. In this phase one trial (NCT03215810), patients with metastatic NSCLC who progressed after nivolumab treatment were treated with TILs. Patients received the infusion of TILs and IL-2, followed by nivolumab to augment the persistence of the TILs. Two of 13 evaluable patients achieved durable complete responses. The exciting results of this clinical trial offer hope for patients who are progressing after anti-PD-1 treatment, and indicate that TIL therapy combined with a PD-1 inhibitor may be a promising choice for patients with metastatic NSCLC. Currently, a clinical trial using ACT with TIL for the treatment of NSCLC is recruiting patients (NCT04614103), and several trials of TIL combined with PD-1 inhibitor for the treatment of NSCLC have been launched (NCT03903887, NCT03645928).

## 4. Preparation of TIL

**Figure 1:**
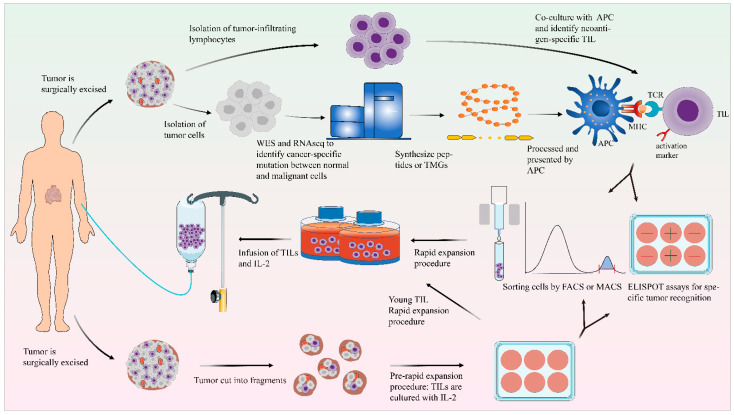
General scheme of the preparation of TILs. After excision, the tumor tissue is fragmented or digested into a single-cell suspension and then cultured in cell culture plates with IL-2. Using the selected TIL method, the detection of tumor reactivity is based on the production of IFN-γ by co-culturing with autologous tumor cells. In the “young” TIL method, the assays for specific tumor recognition are omitted. TILs could be enriched by FACS or MACS. TIL cultures are then expanded to treatment levels by REP. These TILs are then infused back into the lymphodepleted patient. On the other side, the tumor cells and normal cells undergo WES and RNA-seq to identify mutations. Based on this information, TMG or peptides are synthesized. These TMG or peptides are then processed by autologous APC and presented to T cells which are co-cultured with APCs. The recognition of neoantigen-specific T cells depends on IFN-γ ELISPOT assays, and these identified T cells with activated surface markers (such as CD137) can be purified by FACS.

Although TILs exist largely in the tumor microenvironment, this does not mean TILs can be successfully isolated and amplified from tumor tissues, nor does it mean tumor cells can be recognized and eliminated by isolated TILs. The ability of TILs to kill tumor cells will be inhibited by various factors in the tumor microenvironment, such as the existence of immunosuppressive subsets including regulatory T cells (Tregs), myeloid-derived suppressor cells (MDSCs), and tumor-associated macrophages, as well as the increase in immunosuppressive molecules and metabolites [22,23]. Furthermore, the number of TILs in the tumor microenvironment is small and difficult to amplify. Therefore, it is essential to optimize the preparation process of TILs. In general, the generation of TILs can be divided into two steps: the pre-rapid expansion procedure (pre-REP), which is the initial growth stage of TILs from tumor tissue, and the large-scale expansion of the 14-day REP (Figure 1).

### 4.1. Pre-Rapid Expansion Procedure

Generally, fresh tumor tissue is obtained through surgery, and should be processed in a reasonable amount of time. For initial growth of TILs, tumor tissue can be cut into fragments or made into a single-cell suspension through enzyme digestion, physical disaggregation, or fine needle aspiration [24,25]. The tumor fragments or single-cell suspensions are then cultured in a complete medium with IL-2.

The tumor tissues for initial growth of TILs may be from breast [7], skin [6], metastatic lymph nodes [26], lung [11], colorectum [10], or other body parts. It is important to note that the tumor location may affect the production of TILs. TILs from the colorectal or skin regions may be contaminated with microbes, lymph nodes may contain a large number of ineffective T cells, and the fibroblasts may occupy the whole culture container [27].

### 4.2. Unselected/Young TILs

In most early studies, only the tumor-reactive cultures that detected interferon-gamma (IFN-γ) in co-culture with autologous tumor materials or tumor cells in vitro could be pre-selected for further outgrowth. However, this “selected” method largely limits the clinical application of TIL therapy, which adds to the time required for the TIL expansion, and the tumor cells or materials used for testing are difficult to obtain [28,29]. Remarkably, several studies showed that a shorter culture duration before beginning the REP is beneficial to clinical response [29,30,31].

In order to simplify the generation process of TILs and potentially improve the properties of TIL cultures, a modified TIL production protocol which uses “unselected” TILs to start the REP protocol was tested [32,33]. These unselected bulk TILs, called “young” TILs, have no pre-selection step for tumor reactivity based on IFN-γ (Figure 1). Crucially, the clinical response rate of the protocols which use “young” TILs is not low [34], which means the status of “young” TIL schemes are rapidly improving [35]. Studies demonstrated that “young” TILs have longer telomeres and higher levels of the costimulatory molecules CD27 and CD28 compared with the selected TILs, which means “young” TILs at a low level of differentiation are conducive to the proliferation, survival, and persistence of TILs in vivo [32,36].

### 4.3. TIL Selection Strategies

The TILs isolated from the complex tumor microenvironment are a heterogeneous cellular population. The effector T cells with anti-tumor activity are required, so it is necessary to screen the effector T cells. Isolation strategies have been proposed and developed for this program. Here, we attempt to summarize the known strategies for isolating tumor-reactive T cells.

#### 4.3.1. Selection by Surface Markers

TILs could be enriched through selection markers (Figure 1). CD8^+^ TILs play a key role in immune response; they can specifically recognize and kill tumor cells [37], although CD4^+^ T cells also show the potential to kill tumors [9]. It is reported that the infusion of a large amount of CD8^+^ TILs is associated with an objective clinical response [34,38]. Therefore, the enrichment of CD8^+^ TILs is a feasible method to enhance tumor reactivity.

Studies have shown that CD103^+^ TILs indicate strong prognostic significance in ovarian and oral cancer [39,40]. In 2017, Ganesan et al. demonstrated that CD103 and CD8 are important phenotypes in the tissue-resident memory of T cells in lung cancers [41]. Furthermore, Simoni et al. reported that CD39 could be a marker for recognizing tumor-reactive CD8^+^ T cells [42]. In 2018, Doohen et al. proved that CD103^+^, CD39^+^, and CD8 TILs are unique tumor-reactive cells in the tumor microenvironment, which can be isolated from the tumor digests by immunomagnetic beads and fluorescence-activated cell sorting (FACS) [43]. The co-culture experiment of TILs and tumor cells showed that CD103^+^, CD39^+^, and CD8 TILs exhibited enhanced tumor reactivity [43]. In 2020, Kortekaas and his colleagues highlighted that CD39^+^ T cells have specific tumor reactivity, and have further proven the importance of CD39 in identifying and isolating tumor-reactive T cells [44]. However, CD39 was also highly expressed on exhausted CD8 TILs with low proliferation potential [45], and in order to improve the proliferation potential of tumor-specific CD8 TILs a new sorting strategy with negative CD39 was defined [45].

Although PD-1/CD279 has been reported as an immunosuppressive checkpoint, which is expressed on activated T cells and hijacked by tumors to escape immune surveillance and induce immune tolerance [46,47,48], it could be more accurately defined as a marker for tumor-reactive T cells to recognize tumor cells [49]. Inozume et al. pre-selected CD8^+^ PD-1^+^ T cells using the FACS method and immunomagnetic beads, and these cells showed greater tumor reactivity when compared to CD8^+^ PD-1^−^ or non-selected TILs [50]. Fernandez-Poma et al. [51] found that the expression of PD-1 on CD8+ TILs accurately defined the tumor-reactive cells and evaluated the antitumor activity of CD8^+^ PD-1^+^ TILs in vivo, which provide direct support for this notion.

Current strategies also include selection by the co-stimulatory marker 4-1BB/CD137, which can be detected on all phenotypes of activated CD8^+^ T cells [52] and natural tumor-reactive TILs [53]. CD137, a costimulatory molecule, is induced by specific interactions between T cells and tumor cells [54,55]. The stimulation of tumor antigens can upregulate the expression of CD137 on T cells and mediates the activation and proliferation of T cells [53,56]. The screening scheme based on CD137 is widely advocated because it can recognize a wide range of antigens, including new antigens and shared tumor antigens [55]. Studies have shown that CD8^+^ TILs expressing 4-1BB seems to represent tumor-specific T cell subsets of melanoma [53,57]. Poch et al. [26] and Tavera et al. [58] proposed an amplification method of TILs co-stimulated by CD137 and IL-2, which rapidly increased the amplification rate of TILs in skin, uveal melanoma, and primary bladder cancer. However, as CD137 is a costimulatory molecule, long-term stimulation makes it over-differentiated, and its proliferative potential is affected. Parkhurst et al. proposed a strategy, which screen upregulates CD137 after antigen-specific stimulation to enrich tumor-reactive T cells and isolate TCRs, and then introduce it into less-differentiated PBLs for reinfusion therapy [59]. However, for most advanced metastatic patients, a lengthy preparation time is unreasonable. Seliktar-Ofir et al. proposed a simplified and efficient protocol. Briefly, the CD137 positive TIL was directly isolated from the co-culture system of TILs and tumor cells, and then the standard rapid amplification steps were carried out. TCR sequencing steps were omitted [55].

#### 4.3.2. Selection by Neoantigens

TIL therapy depends largely on the recognition of specific tumor antigens, especially neoantigens. To this end, Rosenberg and his colleagues combined whole-exome sequencing (WES) technology and human leucocyte antigen (HLA) class I to identify and candidate the mutant proteins expressed in tumor cells that differ from healthy cells. These mutated epitopes are then synthesized and introduced to MHC matched antigen-presenting cells (APC). They are then co-cultured with the initial TIL, and the recognition ability of TILs was evaluated by performing ELISPOT assays of IFN-γ [60]. The identified neoantigen-specific CD8^+^ T cells can be further purified by FACS based on activated markers, such as CD137 [59], and then expanded in vitro. With the improvement of the tandem minigene (TMG) library, the ability to recognize and identify neoantigen-specific TILs has been further improved, because it omits the step of identifying and synthesizing a large number of peptides [20] (Figure 1). Using these techniques, neoantigen-specific effector TILs have been successfully performed in patients with melanoma, cholangiocarcinoma, colorectal cancer, breast cancer, and ovarian cancer [7,9,10,12,56]. However, this method also takes a lot of time to prepare the TIL for reinfusion, and requires complex equipment, making this unsuitable for most advanced patients. In addition, the proliferative potential of TILs will be also impaired by long-term antigen stimulation and amplification. Recently, Deniger and colleagues transferred the TP53 “hot spot” mutation-reactive T cell receptor to peripheral blood T cells, which may provide a potential solution [12].

### 4.4. Rapid Expansion Procedure

When an adequate quantity of the initial TIL has been obtained, it is then immediately cryopreserved or used directly for further REP, which usually takes approximately 14 days in the standard protocol. During the REP, TILs are stimulated and further expanded to treatment levels with IL-2, anti-CD3 antibody (which is added only at the start of REP), and irradiated feeder cells. The irradiated feeder cells originate from allogeneic or autologous peripheral blood mononuclear cells, which can activate and release growth factors to promote the expansion of TILs. During the REP, TILs are usually cultured in a culture flask and then transferred to gas-permeable bags to expand the cultures. In order to improve the growth rates of TILs, Jin et al. used gas-permeable flasks for the initial growth and REP of TILs [61].

## 5. Role of Lymphodepletion

A non-myeloablative (NMA) lymphodepletion regimen with chemotherapy or total body irradiation (TBI) before TIL infusion is an important milestone in TIL therapy. The application of the lymphodepletion regimen comes from a murine model in which the efficiency of TILs is improved after lymphodepletion [3]. It is reported that the lymphodepletion regimen can enhance the effect of TILs through several potential mechanisms, such as the elimination of Tregs, by increasing host homeostatic cytokines (including IL-7 and IL-15), and by decreasing endogenous lymphocytes, which compete for these trophic cytokines [62,63]. Moreover, lymphodepleting can enhance the activation of antigen presenting cells (APC) which play an important role in regulating adoptively transferred T cells [64].

In 1994, Rosenberg et al. showed that the ORR in patients with metastatic melanoma treated with and without lymphodepletion were 35% and 31%, respectively [65]. Similarly, in 2002, Dudley and his colleagues demonstrated that the adoptive transfer of TILs could achieve a better ORR after lymphodepletion of cyclophosphamide (Cy) 60 mg/kg/day for two days and fludarabine (Flu) 25 mg/m^2^/day for five days in patients with metastatic melanoma [66]. Some clinical studies suggested that adding TBI to the NMA chemotherapy regimen could improve the response rate [6,67]. However, there was no obvious effect in a randomized trial [68]. Recently, a study summarized different NMA regimens, and suggested that the regimen of Cy (30 mg/kg for two days) and Flu (25 mg/m^2^ for five days) is a better choice [69], which has maximum effect on lymphodepletion with minimum toxicity compared to other doses and regimens. Notably, most patients have experienced adverse effects caused by the NMA lymphodepletion regimen, such as neutropenia, lymphopenia, and coagulopathy, which are hematological side effects [5,70]. Non-hematological events include nausea, headaches, loss of appetite, neutropenic fever, diarrhea, and hyperbilirubinemia [5,69], which are mostly manageable by standard supportive treatment. Interestingly, in order to avoid the severe toxicity of the NMA lymphodepletion regimen, Santos et al. designed a regimen which used oncolytic adenoviruses to replace NMA lymphodepleting which resulted in great efficacy [71].

## 6. Role of Interleukin-2

IL-2 is an important cytokine; it stimulates effector T cell growth and survival [72]. Rosenberg and his colleagues found that the addition of IL-2 to cultured lymphocytes promoted lymphocytes to lyse autologous tumor cells [73,74]. In addition, they found that the combination of IL-2 with an infusion of TILs could improve their therapeutic effect [3]. Rosenberg then applied TIL therapy combined with IL-2 to patients with melanoma and published the first report on the successful treatment of melanoma with TIL-ACT in 1988 [4].

However, it is reported that IL-2 is an essential cytokine for the development and function of Tregs [75,76], which would compete with CD8^+^ T cells for IL-2 and suppress the response of the CD8^+^ T cells [76]. Liu et al. have proved that IL-2 could regulate tumor-reactive CD8^+^ T cell exhaustion [77]. Briefly, high levels of IL-2 upregulate the inhibitory receptors of CD8^+^ T cells and reduce the production of cytokines and effector molecules, resulting in the dysfunction of CD8^+^ T cells [77]. IL-2 could lead to terminal differentiation and T cell exhaustion, while IL-7, IL-15, and IL-21 can produce poorly differentiated T cells and enhanced proliferation [78]. Compared with the use of IL-2 alone, the addition of cytokines IL-15 and IL-21 to the culture medium could increase the number of TILs cultured from lung and colorectal tumors [79,80,81]. However, the current clinical trials mainly use IL-2 to expand and maintain TILs, and further clinical trials are needed to determine whether these cytokines can replace IL-2.

The optimal dose of IL-2 infusion is yet to be determined. Since Rosenberg and his colleagues published their research, the regimen of TILs combined with IL-2 has been used in the treatment of patients with metastatic melanoma for decades [6]. It has been reported that the HD IL-2 (≥720,000 IU/kg) regimen, which when administrated after TIL infusion, can continuously support the growth and activity of infused TILs [68]. However, HD IL-2 is associated with severe adverse events, include capillary leakage syndrome, which is characterized by hypotension, oliguria, edema, and even a hypovolemic shock [82,83]. Therefore, LD IL-2 (<720,000 IU/kg) combined with a TIL infusion is worthy of investigation. A study from Copenhagen demonstrated that LD IL-2 combined with a TIL infusion resulted in two out of six patients with metastatic melanoma having durable objective responses [84]. Furthermore, Ullenhag et al. showed that five out of 24 patients had objective clinical responses after LD IL-2 treatment [85]. In another clinical trial, the ORR of the patients who received attenuated doses of IL2 was 42% [86]. Recently, in a phase two clinical trial, 12 patients treated with TILs followed by a LD IL-2 regimen (125,000 IU/kg/day over 12 days) experienced no grade five IL-2-related adverse events, but did show the expected blood toxicities caused by lymphodepletion. Only three patients had a partial clinical response, and no patients achieved a complete response. Nguyen et al. believes that this unsatisfactory result may be caused by the LD IL-2 regimen [87].

There is not yet sufficient evidence to suggest that LD IL-2 can achieve a sustained response comparable to that of HD IL-2 regimens. However, these results are promising; they must be urgently addressed and further studied in clinical trials (Table 1). Recently, Hsu et al. have engineered an IL-2 prodrug to solve the problem of high toxicity in vivo and short half-life [88].

## 7. Combination Therapy with TILs

### 7.1. Immune Checkpoint Inhibitors

The combination of TIL therapy and anti-PD-1/PD-L1 antibody therapy has shown preliminary favorable results in some recent trials [11,89]. The immune checkpoint receptors (e.g., CTLA-4 and PD-1/PD-L1) are expressed on the surface of T cells, which is the self-protective mechanism of the immune system. In cancer patients, the CTLA-4 and PD-1 molecules on effector T cells are upregulated and, respectively, bind to B7-1/B7-2 and PD-L1 of antigen-presenting cells or tumor cells. This results in inhibited T cell function, which can be blocked by anti-CTLA-4 and anti-PD-1 antibodies [90]. In addition, studies have demonstrated that after long-term exposure to tumor antigens, CD8^+^ T cells will show apoptosis, or enter a state of abnormal differentiation with high express inhibitory receptors and almost no response to specific tumor antigens, which could be remedied with a checkpoint inhibitor [91,92,93]. In addition, the in vivo treatment models also proved these results [94,95]. These mechanisms provide a theoretical basis for TILs combined with ICIs. Therefore, to obtain increased tumor-reactive TILs, the ICIs should be administrated before the resection of tumor tissues and during the initial growth of TILs [96,97,98,99]. In order to make infused TILs more tumoricidal, ICIs were also administered after TIL infusion [5,7,11,97]. The effect of TILs combined with anti-PD-1 therapy as a first-line treatment is still in the clinical trial stage, and the additive effect of TILs and anti-PD-1 cannot be evaluated until the preliminary results are released. Recent studies have found that in addition to tumor cells, dendritic cells (DC) also express high levels of PD-L1, which can weaken the activation of T cells and inhibit anti-tumor activity [100,101]. This will provide a theoretical basis for TILs combined with PD-L1 inhibitors in the treatment of cancer patients with high PD-L1 expression.

### 7.2. BRAF Inhibitor

**Figure 2:**
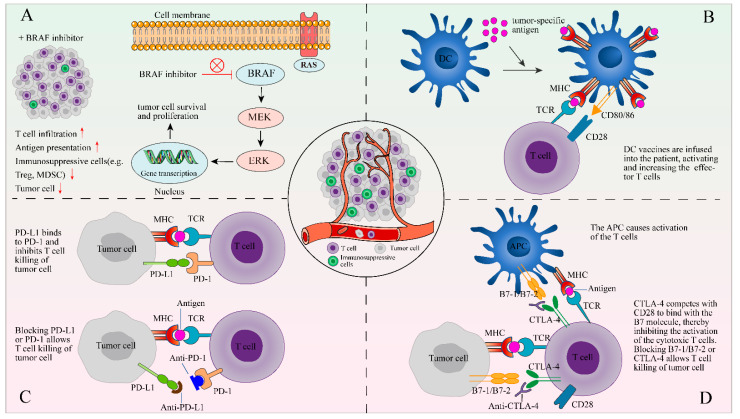
The combination of TIL therapy and other therapies. The following four methods of combined therapy are described in the form of figures: (**A**) a BRAF inhibitor can reduce immunosuppressive signals and facilitate tumor infiltration of lymphocytes; (**B**) DCs are stimulated by signals and loaded with tumor-specific antigens on MHC to activate the antigen-specific T-cells; (**C**) PD-L1 or PD-1 blockers may prevent the interaction of PD-1 and PDL-1 between TILs and tumor cells; and (**D**) blocking CTLA-4, which competes with CD28 to bind the B7 molecule, allows T cell killing of tumor cell.

The BRAF gene plays an important role in cell growth and differentiation. In some cancers, BRAF mutations change the cascade of ERK/MAPK signals, resulting in increased cell proliferation [102]. BRAF mutation is the most common mutation that causes overactivation of the MAPK pathway and has been found to carry it in approximately half of cutaneous melanomas [103]. The activating BRAF mutation (Mainly V600E) can induce immune-escape mechanisms, which make them “dull” in immunity and gives them the ability to evade T-cell immune responses [104]. Studies have shown that the BRAF inhibitor vemurafenib can reduce related immunosuppressive signals, facilitate infiltration of lymphocytes, and reduce immunosuppressive cells, as well as enhance the presentation of melanoma antigens [105,106] (Figure 2). The ORR of BRAF inhibitor Vemurafenib in the treatment of BRAF^V600E^ mutant melanoma is up to 50%, which improves the progression-free and overall survival rate [107]. However, the clinical response of BRAF inhibitors is usually short-lived [108], and as the disease progresses, alternative treatments available to patients is limited. Peiffer and colleagues indicated that BRAF/MEK inhibitors can promote the proliferation of melanoma-specific T cells in patients with melanoma [109]. Recently, in a clinical trial, seven of 11 patients with metastatic melanoma who received a combination treatment of TILs, HD IL-2, and vemurafenib experienced an objective clinical response, two of which achieved a complete response [110].

### 7.3. Other Combination Therapy with TILs

Notably, the DC vaccine can induce an immune response and can activate and increase the number of TILs [111,112] (Figure 2), and its combination with TIL therapy is being evaluated in clinical trials (NCT01946373, Table 1). The combination of TIL therapy and oncolytic virus is also being explored [113,114]. The virus can combat tumor immunosuppression by producing cytokines that promote the anti-tumor effect of TILs [115]. In a clinical trial of TIL therapy combined with adenovirus in the treatment of metastatic melanoma, five of 13 patients achieved objective responses, of which three achieved complete responses [115].

## 8. Limitations of TIL Therapy and Future Perspectives

TIL therapy has been successfully applied to patients with metastatic melanoma and other solid tumors. Compared with other ACTs (such as CAR-T and TCR-T), TIL therapy has its unique advantages. TILs are composed of T cells with multiple TCR clones, which can not only directly act against shared self-antigens [116], but can also act against tumor-specific neoantigens [117], making it more effective in response to tumor heterogeneity. TILs usually contain a large number of effector memory T cells and express chemokine receptors after being stimulated by the tumor antigen in vivo, which makes it easier to locate in the tumor tissue after transfusion. In addition, TILs come from patients themselves without gene modification, meaning the method has low toxicity.

However, TIL therapy obviously also has its limitations. Firstly, in order to achieve durable anti-tumor responses, effector T cells with anti-tumor activity must be present in the tumor, which is not the case for many solid tumors. In contrast, the NK cells have higher feasibility for manufacturing. However, NK cells are generally inhibited in cancer patients and are easily affected by a series of inhibitory factors in the cancer microenvironment [118,119]. Another potential translational anti-tumor cell population are tumor infiltrating γδT cells, which usually perform their anti-tumor activity by secreting interferon (IFN)γ and tumor necrotic factor (TNF), but they are not dependent on tumor-associated antigens [120]. To overcome the homing challenges of γδT cells, many strategies, including bispecific molecules [121], topical injection [122], and combination use with checkpoint inhibitors [123], have been tried for solid tumor treatment.

Secondly, although the selection strategies of TILs have made great progress, due to the inability to efficiently identify and isolate neoantigen-specific lymphocytes, as well as the obstacle of the immunosuppressive tumor microenvironment, the wide application of TIL therapy in a variety of cancers is still a challenge. Unlike TIL therapy, T cell receptor-engineered T cell (TCR-T) therapy modifies the TCRs on the surface of endogenous T cells to recognize tumor-specific neoantigens [124]. Therefore, TCR-T therapy is free from the limit of surface antigen expression of the target cells, which is a potential cellular immunotherapy for the treatment of various cancers. Significantly, the TCRs that respond to TP53 mutations identified by Deniger et al. may provide a new treatment for patients who carry these mutations and express the corresponding HLA types [12]. Moreover, as CAR-T cells are not required in APC presentation to act against antigen-positive cancer cells [125], CAR technology is a potential strategy for modifying TILs to recognize tumor associated antigens or neoantigens and enhance the therapy effect of TILs. A recent study by Mills et.al. designed dual-specific TILs by transducing a CAR to act against the common tumor antigen Her2 (anti-Her2 CAR-TILs); further studies showed that anti-Her2 CAR-TILs could act against Her-2 positive tumors in vitro and in vivo [126]. However, as tumor cells usually show different types of gene mutations which will generate many kinds of neoantigens, so it is difficult to design universal CARs-TILs to eliminate cancer cells.

Meanwhile, the immunosuppressive tumor microenvironment may induce the exhaustion of infiltrating cytotoxic T cells, which in turn results in reducing the elimination ability of cancer cells. Thus, new specific exhaustion markers must be explored. The development of new technologies, such as single-cell level analytic technologies, could help to unravel the characteristics of heterogeneous TILs; new TIL subsets could be identified with therapeutic potentials or new T cell markers as potential targets to act against solid cancers [127]. Using single-cell RNA sequencing, Singer et.al. separated the activation and dysfunction gene modules in dysfunctional CD8^+^ TILs, further investigation showed that the zinc-finger transcription factor Gata-3 is a regulator of CD8^+^ TILs dysfunction [128]. Using single-cell mass cytometry, Wagner et al. found high-grade ER^+^ breast tumors infiltrated with high levels of PD-L1^+^ exhausted T cells [129].

In addition, the infused TIL has a short survival time in vivo. In order to improve the survival and tumor-homing ability of TILs after reinfusion into patients, the modification technology of TILs has been explored [130,131]. Remarkably, researchers from the Jean-Philippe Girard research group of the University of Toulouse in France have found that the tumor-associated high endothelial venules (TA-HEVs) are the main sites that mediate the entry of lymphocytes into tumors. Increasing the density and maturity of TA-HEV endothelial cells (TA-HECs) can promote tumor-specific CD8+T cell infiltration and improve the efficacy of ICI. Moreover, TA-HEVs are closely related to the efficacy of ICI in patients with melanoma, and can be used as a powerful predictor of clinical treatment [132] and a potential target of combined TIL therapy.

## 9. Conclusions

In conclusion, TIL therapy has some unique advantages in the treatment of solid tumors, but it still faces a series of challenges. The tumor immunosuppressive microenvironment is still the main obstacle of TIL therapy. Furthermore, there is still considerable room for improvement in the isolation and expansion of effective tumor-reactive T cells, and alternative combined therapies still need to be explored.

## Figures and Tables

**Table 1:** Clinical trials with TILs for melanoma from *ClinicalTrials.gov* between 2011 and 2021.

Identification Number	Status	Phase	Study Start	Lymphodepletion Regimen	TIL Product	IL-2 Regimen	Combination Therapy
NCT04812470	Not yet recruiting	I	1 November 2021	Melphalan i.v. for once	Not described	s.c. q.d. for up to 14 d	/
NCT05050006	Recruiting	II	October 2021	Cy and Flu for 5 d	Not described	up to 8 doses	/
NCT05098184	Recruiting	Early I	26 September 2021	Cy and Flu	1 × 10^9^ to 5 × 10^10^ TILs	Not described	/
NCT04924413	Not yet recruiting	II	1 July 2021	/	L-TIL (3–10) × 10^9^/m^2^/cycle for 4 cycles	/	Tisleli 200 mg, i.v. q.3 w
NCT03991741	Recruiting	I	7 October 2020	Not described	Not described	720,000 IU/kg t.i.d, for up to 15 doses	/
NCT04165967	Recruiting	I	17 September 2020	Not described	5 × 10^9^–2 × 10^11^ TILs	125,000 IU/kg/day s.c. for max 12 d	start at d 14: Nivo (240 mg i.v. every 2 w max 24 months)
NCT04223648	Not yet recruiting	Early I	February 2020	Not described	PD-1^+^ TILs	Not described	1 cycle of Tremeli/Durva before tumor resection, 3 cycles of Ipili/Nivo after TIL infusion, followed by Nivo
NCT03645928	Recruiting	II	7 May 2019	Not described	Experimental 1 A: LN-144 TIL; Experimental 1 B: LN-145-S1 TIL; Experimental 1 C: LN-144 TIL	Not described	Experimental 1 A: Pembro administered following tumor resection (q 3 w or q 6 w until 24 months)
NCT03467516	Recruiting	II	14 May 2018	Cy and Flu	1 ×10^9^–2 × 10^11^ TIL per current standard protocol	600,000 IU/kg t.i.d. max 6 doses	/
NCT03374839	Recruiting	I/II	12 February 2018	Not described	Experimental 1: 5 × 10^8^ TIL (3 patients) Experimental 2: 1–20 × 10^9^ TIL at 14 w and 18 w	600,000 IU/kg/d for 5 d	Nivo (from day 0.3 mg/kg every 2 w until w 52)
NCT03475134	Recruiting	I	21 February 2018	Cy i.v. for 2 d and Flu i.v. 5 d	Not described	HD t.i.d. max 8 doses	Nivo (first year: 240 mg, every 2 w; second year: 480 mg every 4 w)
NCT03166397	Recruiting	II	5 June 2017	Cy (30 mg/kg/d for 2 d) + Flu (25 mg/m^2^ for 3 d)	Not described	720,000 IU/kg t.i.d, max 10 doses	/
NCT03158935	Completed	I	7 July 2017	Experimental 1: Cy i.v. 60 mg/kg/d for 2 d + Flu 25 mg/m^2^ for 5 d	1 × 10^10^ to 1.6 × 10^11^ TILs	Experimental 1 + 2: 125,000 IU/kg s.c./d	Experimental 1: Pembro (200 mg q 3 w)
NCT02652455	Active, not recruiting	Early I	8 March 2016	Cy 2 d beginning 3–6 w after tumor collection for TIL growth + Flu for 5 d	Not described, outgrowth in 4–8 w with CD137 activating antibody	Not described	treatment with Nivo before tumor resection; anti-CD137 agonistic antibody culture TIL
NCT02621021	Recruiting	II	4 December 2015	Experimental 1 + 2: Cy 60 mg/kg i.v. for 2 d + Flu 25 mg/m^2^ for 5 d	Young TIL	Experimental 1 + 2: 720,000 IU/kg i.v. t.i.d., max 12 doses	Experimental 1 and 2: Pembro 2 mg/kg i.v. on d −2, d 21 (+/− 2 d), 42 (+/− 2 d), and 63 (+/− 2 d)
NCT02360579	Active, not recruiting	II	September 2015	Lymphodepleting chemotherapy	Experimental 1: LN-144 autologous TIL noncryopreserved product Experimental 2: LN-144 autologous TIL cryopreserved Experimental 3: LN-144 autologous TIL retreatment for 2nd LN-144 infusion	Not described	/
NCT01740557	Recruiting	I/II	28 January 2015	Cyc 60 mg/kg for 2 d + Flu 25 mg/m^2^ for 5 d	Up to 1.5 × 10^11^ TIL (CXCR2 and NGFR transduced TIL)	720,000 IU/kgi.v. every 8–16 h, max 15 doses	/
NCT02354690	Completed	I/II	November 2014	Cy 60 mg/kg for 2 d + Flu 25 mg/m^2^ for 5 d	4–6 w culture time Infusion 1 × 10^9^–2 × 10^11^ TILs	Decrescendo regimen (18 MIU/m^2^ for 6 h, 18 MIU/m^2^ for 12 h, 18 MIU/m^2^ for 24 h followed by 4.5 MIU/m^2^ for another 3 × 24 h)	Pre-treatment with Vem 960 b.i.d. 7 d before tumor harvest until lymphodepletion
NCT02379195	Completed	I/II	November 2014	Cy 60 mg/kg i.v for 2 d + Flu 25 mg/m^2^ i.v for 5 d	4–6 w culture time Maximum number of TILs	Continuous infusion decrescendo regimen (18 MIU/m^2^ IL-2 over 6 h, 18 MIU/m^2^ IL-2 over 12 h, 18 MIU/m^2^ IL-2 over 24 h followed by 4.5 MIU/m^2^ IL-2 over 24 h for 3 d	s.c. injections of Peginterferon alfa-2b 3 microgram/kg (d −2, d 7 and d 14)
NCT01955460	Recruiting	I	15 October 2014	Cy 60 mg/kg i.v. for 2 d + Flu 25 mg/m^2^ i.v. for 5 d	Transduced DNRII TIL, equal number of transduced NGFR TIL, up to a total of 1.5 × 10^11^ TIL	720,000 IU/kg i.v. every 8–16 h max 15 doses on d 1–5 + 22–26	/
NCT02278887	Recruiting	III	September 2014	Cy 60 mg/kg iv for 2 d + Flu 25 mg/m^2^ for 5 d	Not described	600,000 IU/kg t.i.d., max 15 doses	/
NCT01993719	Active, not recruiting	II	12 December 2013	Cy 60 mg/kg/day for 2 d + Flu 25 mg/m^2^ i.v. for 5 d	Young TIL	720,000 IU/kg i.v. t.i.d., max 12 doses	Experimental 1: Pembro 2 mg/kg IV on Days −2, 21 (+/− 2 days), 42 (+/− 2 days), and 63 (+/− 2 days)
NCT01946373	Recruiting	I	October 2013	Cy 60 mg/kg i.v. (d −7 to −6) + Flu 25 mg/m^2^ i.v. (d −5 to −1)	Up to 5 × 10^10^ TILs i.v. infusion	100,000 IU/kg t.i.d., maximum 14 doses	Experimental 2: i.d. DC vaccinations with up to 1.5×10^7^ DC pulsed with autologous tumor lysate and NY-ESO-1 peptide after completion of IL-2
NCT01995344	Unknown	II	October 2013	Cy 60 mg/kg 2 d + Flu 25 mg/m^2^ 5 d	Not described	Arm A: HD IL-2, max 12 doses Arm B: LD IL-2, max 12 doses	/
NCT01807182	Active, not recruiting	II	20 August 2013	Cy for 2 d + Flu for 5 d	Not described	Not described	/
NCT01883323	Completed	II	June 2013	Cy 60 mg/kg i.v. for 2 d + Flu 25 mg/m^2^ i.v. for 5 d	1 × 10^10^–1.6 × 10^11^ TILs	125,000 IU/kg/d for 2 w (2 d rest between each w)	/
NCT01701674	Active, not recruiting	Not Applicable	9 October 2012	Cy for 2 d + Flu for 5 d	Not described	HD IL-2 t.i.d., max 15 doses	cycle 1: Pre-treatment with Ipili prior to surgery. Cycle 2 of Ipili 1 w after surgery (3 w after 1st cycle)
NCT01659151	Active, not recruiting	II	3 August 2012	Cy for 2 d + Flu for 5 d	Not described	HD IL-2	Pre-treatment with Vem (3 w prior to TIL + post TIL up to 2 yr)
NCT01319565	Active, not recruiting	II	24 March 2011	Experimental 1 + 2: Cy 60 mg/kg for 2 d + Flu 25 mg/m^2^ for 5 d;Experimental 2: d −3 to −1,2 Gy of TBI for 3 d (total dose 12 Gy)	1 × 10^9^–2 × 10^11^ young TILs	720,000 IU/kg i.v. t.i.d., max 15 doses	/

Abbreviations: s.c., subcutaneous; t.i.d., ter in die; qd, quaque die; b.i.d., bis in die; CXCR, C-X-C chemokine receptor; Cy, cyclophosphamide; d, day; flu, fludarabine; Gy, Gray; HD, high dose; hr, hour; i.d., intradermal; i.v., intravenous; IL-2, interleukin-2; IU, international unit; kg, kilogram; LD, low dose; max, maximum; mg, milligram; NGFR, nerve growth factor receptor; nivo, nivolumab; ipili, ipilimumab; tremeli, tremelimumab; durva, durvalumab; tisleli, tislelizumab; PD-1, programmed cell death protein-1; PD-L1, programmed death ligand-1; pembro, pembrolizumab; TBI, total body irradiation; TIL, tumor-infiltrating lymphocytes; w, week; x, times; vem, vemurafenib; DC, dendritic cells.

**Table 2:** Recent preliminary studies have shown that TIL therapy is effective in patients with other solid tumors.

Cancer Type	Lymphodepletion Regimen	TIL Product	IL-2 Regimen	Combination Therapy	ORR	CR
HPV-associated carcinomas	Cy and Flu	HPV-TILs	720,000 IU/kg,t.i.d.	/	24%	6%
Cervical cancer	Cy and Flu	HPV-TILs	720,000 IU/kg,t.i.d.	/	33%	22%
Cervical cancer	Cy and Flu	LN-145, mean number of 2.8 × 10^10^	600,000 IU/kg	/	44%	3.7%
Colorectal cancer	Cy and Flu	1.11 × 10^11^ TILs, reactive to mutant KRAS G12D	720,000 IU/kg	/	/	/
cholangiocarcinoma	Cy and Flu	4.24 × 10^10^ and 1.26 × 10^11^ TILs (ERBB2IP mutation-reactive T cells)	720,000 IU/kg	/	/	/
non-small cell lung cancer	Cy and Flu	median number of 9.5 × 10^10^	intermediate-dose decrescendo IL-2	Nivo 240 mg for 4 doses prior to TIL, 480 mg up to 12 months after TIL	/	12.5%
breast cancer	Cy and Flu	8.2 × 10^10^ TILs	720,000 IU/kg,t.i.d.	pembro 2 mg/kg (d −2, d 21, d 42 and d 63)	/	/

Abbreviations: t.i.d., ter in die; cy, cyclophosphamide; d, day; flu, fludarabine; IL-2, interleukin-2; mg, milligram; nivo, nivolumab; pembro, pembrolizumab; TIL, tumor-infiltrating lymphocytes; ORR, objective response rate; CR, complete response.

## Abbreviations

ACT: adoptive cell therapy; APC: antigen presenting cell; BRAF: v-raf murine sarcoma viral oncogene homolog B1; b.i.d.: bis in die; CD: cluster of differentiation; Cy: cyclophosphamide; d: day; CXCR: c-x-c chemokine receptor; CRR: complete response rate; CTLA-4: cytotoxic T lymphocyte associated antigen-4; DC: dendritic cells; Durva: durvalumab; FACS: fluorescence-activated cell sorting; flu: fludarabine; FDA: food and drug administration; gy: gray; HD: high dose; HPVs: human papilloma viruses; hr: hour; i.d.: intradermal; i.v.: intravenous; IU: international unit; IL-2: interleukin-2; IFN-γ: interferon-gamma; ICI: immune checkpoint inhibitors; kg: kilogram; LD: low dose; Ipili: ipilimumab; max: maximum; m: milligram; MHC: histocompatibility complex; NGFR: nerve growth factor receptor; nivo: nivolumab; NMA: non-myeloablative; NK: natural killer; NSCLC: non-small cell lung cancer; ORR: objective response rate; pembro: pembrolizumab; PD-1: programmed cell death protein-1; PD-L1: programmed cell death protein ligand-1; qd.: quaque die; REP: rapid expansion procedure; RNA: ribonucleic acid; RCC: renal cell carcinoma; s.c.: subcutaneous; TMG: tandem minigene; t.i.d.: ter in die; TBI: total body irradiation; tremeli: tremelimumab; TILs: tumor-infiltrating lymphocytes; tregs: regulatory T cells; tisleli: tislelizumab; vem: vemurafenib; VEGF: vascular endothelial growth factor; WES: whole-exome sequencing; w: week; x: times.
